# Oxidative stress in hepatitis C infected end-stage renal disease subjects

**DOI:** 10.1186/1471-2334-6-114

**Published:** 2006-07-14

**Authors:** Mehmet Horoz, Cengiz Bolukbas, Filiz F Bolukbas, Mehmet Aslan, Ahmet O Koylu, Sahbettin Selek, Ozcan Erel

**Affiliations:** 1 Harran University, School of Medicine, Department of Internal Medicine, Sanliurfa, Turkey; 2 Harran University, School of Medicine, Division of Gastroenterology, Sanliurfa, Turkey; 3 Harran University, School of Medicine, Department of Biochemistry, Sanliurfa, Turkey

## Abstract

**Background:**

Both uremia and hepatitis C infection is associated with increased oxidative stress. In the present study, we aimed to find out whether hepatitis C infection has any impact on oxidative stress in hemodialysis subjects.

**Methods:**

Sixteen hepatitis C (+) hemodialysis subjects, 24 hepatitis C negative hemodialysis subjects and 24 healthy subjects were included. Total antioxidant capacity, total peroxide level and oxidative stress index were determined in all subjects.

**Results:**

Total antioxidant capacity was significantly higher in controls than hemodialysis subjects with or without hepatitis C infection (all p < 0.05/3), while total peroxide level and oxidative stress index were significantly lower (all p < 0.05/3). Hepatitis C (-) hemodialysis subjects had higher total antioxidant capacity compared to hepatitis C (+) hemodialysis subjects (all p < 0.05/3). Total peroxide level and oxidative stress index was comparable between hemodialysis subjects with or without hepatitis C infection (p > 0.05/3).

**Conclusion:**

Oxidative stress is increased in both hepatitis C (+) and hepatitis C (-) hemodialysis subjects. However, hepatitis C infection seems to not cause any additional increase in oxidative stress in hemodialysis subjects and it may be partly due to protective effect of dialysis treatment on hepatitis C infection.

## Background

Oxidative stress can be defined as an increase in oxidants and/or a decrease in antioxidant capacity [1], and is being increasingly associated with a wide spectrum of renal diseases [2,3]. Oxidative stress in chronic renal failure (CRF) leads to the oxidation of proteins [4], lipids and carbohydrates [5], and facilitates the formation of advanced glycation and lipoxidation products, and possibly promotes many uremic complications [6,7]. There is good evidence indicating that uremia, in general, is associated with enhanced oxidative stress [8,9], and treatment of uremia with hemodialysis (HD) or peritoneal dialysis (PD) has been suggested to contribute to oxidative stress and reduced antioxidant levels in these patients [10,11].

Several prevalence studies of hepatitis C virus (HCV) infection have been undertaken in HD patients. The prevalence of anti-HCV antibody in HD subjects ranged between 10% and 55% [12]. The natural course of HCV infection in HD patients seems to differ from that in other HCV infected patients. HCV-related liver disease usually runs an indolent course in HD patients [13]. It has been shown that HCV infection itself is also characterized by an increase in free radical formation manifested by increased hepatic and serum levels of products of lipid peroxidation [14,15].

Although aggravated production of toxic oxygen radicals and related compounds has been noted in both HD subjects and subjects with HCV infection, little is known about the oxidative status in HD subjects with HCV infection [16-18] and it needs to be elucidated further.

In the present study, we evaluated the oxidative status in HCV (+) HD subjects, HCV (-) HD subjects and healthy controls via measurement of total antioxidant capacity (TAC) [19] and total peroxide level in plasma samples, and calculation of oxidative stress index (OSI) [20]. Thus, we aimed to find out whether HCV infection has any impact on oxidative stress in HD subjects.

## Methods

### Subjects

Sixteen HCV (+) HD subjects, 24 HCV (-) HD subjects and 24 healthy controls were enrolled in the present cross-sectional study. All HD subjects consisted of patients with end-stage renal disease (ESRD) (creatinine clearance = 5 ml/min/1.73m^2^BS), who underwent HD treatment thrice weekly for 4 hours/day with blood flow rates of 180–200 ml/min and dialysate flow rates of 480–500 ml/min using bicarbonate dialysate on hollow-fiber artificial kidneys. Most HD patients were receiving antihypertensive medications (beta blocker, calcium channel blockers, angiotensin-converting enzyme inhibitors and angiotensin-II type 1 receptor blocker). The patients were selected on the basis of their stable clinical condition over the past 3 months. The study protocol was carried out in accordance with the Helsinki Declaration as revised in 1989. All subjects were informed about the study and the written consent was obtained from each one.

The etiology of ESRD in group 1 and 2 was as follows:

Group 1; interstitial nephritis (n = 4), hypertensive nephrosclerosis (n = 6), chronic glomerulonephritis (n = 3), and shrunken kidney with unknown etiology (n = 3).

Group 2; interstitial nephritis (n = 7), hypertensive nephrosclerosis (n = 4), chronic glomerulonephritis (n = 5), polycystic disease (n = 3), and shrunken kidney with unknown etiology (n = 5).

HCV infection was diagnosed by the positivity of anti-HCV and HCV-RNA for at least 6 months of period. In order to avoid the possibility of false negative results, HCV-RNA detection was performed in the whole HD population including HCV negative HD patients.

### Exclusion criteria

History of alcohol abuse, smoking habit, intravenous (IV) drug abuse, pregnancy, and antioxidant use, fish-oil or iron supplement in the previous month, receiving antiviral and/or interferon therapy for HCV (+) subjects, uncontrolled elevated blood pressure, serum total bilirubin level higher than 2 mg/dL, concomitant chronic hepatitis B or other well known liver diseases such as metabolic or autoimmune disorders and various infectious states of the liver, cryglobulinemia, human immune deficiency virus (HIV) infection, diabetes mellitus, chronic respiratory insufficiency, rheumatoid arthritis, cirrhosis, or malignant tumor.

### Virological studies

Anti-HCV was assayed by micro particle enzyme immunoassay (MEIA) (Abbott axsym system, IL USA). HCV-RNA was determined using real time polymerase chain reaction (RT-PCR) method [(Fluorion HCV QNP 2.1 HCV-RNA quantitative kits, Iontek, Istanbul, Turkey) (BioRad ICycler)]. Upper and lower limit of HCV-RNA levels with RT-PCR were 10^2 ^and 10^7 ^copy/ml, respectively.

### Blood collection

Blood samples were obtained in fasting state. Blood samples from dialysis patients were drawn immediately before HD session. Samples were withdrawn from a cubital vein into heparinised tubes and immediately stored on ice at 4°C. The plasma was then separated from the cells by centrifugation at 900 × *g *for 10 min, and the plasma samples were stored at -80°C until analysis as described elsewhere [19,21].

### Biochemical analysis

Serum uric acid, creatinine and blood urea nitrogen (BUN) level were determined using auto-analyzer.

### Measurement of the total antioxidant status of plasma

The total antioxidant status of the plasma was measured using a novel automated colorimetric measurement method for TAC developed by Erel [19]. In this method the hydroxyl radical, the most potent biological radical, is produced by the Fenton reaction, and reacts with the colorless substrate O-dianisidine to produce the dianisyl radical, which is bright yellowish-brown in color. Upon the addition of a plasma sample, the oxidative reactions initiated by the hydroxyl radicals present in the reaction mix are suppressed by the antioxidant components of the plasma, preventing the color change and thereby providing an effective measure of the total antioxidant capacity of the plasma. The assay results are expressed as mmol Trolox eq./L. Within- and between-batch precision values were lower than 3% [19,22].

### Measurement of total plasma peroxide concentration

The total plasma peroxide concentrations were determined using the ferrous oxidation in xylenol orange (FOX)-2 method [23] with minor modifications [20]. The FOX-2 test system is based on the oxidation of ferrous iron to ferric iron by the various types of peroxides contained in the plasma samples, in the presence of xylenol orange which produces a colored ferric-xylenol orange complex whose absorbance can be measured. The FOX2 reagent was prepared by dissolving ammonium ferrous sulphate (9.8 mg) in 250 mM H_2_SO_4 _(10 ml) to give a final concentration of 250 mM ferrous iron in acid. This solution was then added to 90 ml HPLC-grade methanol containing 79.2 mg butylated hydroxytoluene (BHT). Finally, 7.6 mg xylenol orange was added, with stirring, to make the working reagent (250 mM ammonium ferrous sulphate, 100 mMxylenol orange, 25 mM H_2_SO_4_, and 4 nM BHT, in 90% (v/v) methanol in a final volume of 100 ml). The blank reagent contained all the components of the solution except ferrous sulphate. Aliquots (200 μL) of plasma were mixed with 1.8 ml FOX2 reagent. After incubation at room temperature for 30 min, the vials were centrifuged at 12,000 *g *for 10 min. The absorbance of the supernatant was then determined at 560 nm. The total peroxide content of the plasma samples was determined as a function of the difference in absorbance between the test and blank samples using a solution of H_2_O_2 _as standard. The coefficient of variation for individual plasma samples was less than 5%.

### Oxidative stress index

The ratio percentage of the total peroxide to the total anti-oxidant potential gave the oxidative stress index, an indicator of the degree of oxidative stress [20].

### Statistical analysis

Data were presented as mean ± SD. Qualitative variables were assessed by Chi-square test. Non-parametric continuous variables were compared by the Kruskal-Wallis one-way analysis of variance with posthoc analysis using a Mann-Whitney U test. Parametric variables were compared using Student t test and one-way analysis of variance with post-hoc analysis using the Tukey test. Pearson's correlation analysis was used to find out the relationship of alanine aminotransferase with HCV-RNA level in HCV (+) HD subjects. Multiple linear regression analysis was used to find out the relationship of age, gender, dialysis duration, HCV positivity, uric acid, BUN and creatinine with oxidative stress markers. Differences were regarded as significant at 0.05/3 for comparisons were made by Kruskal-Wallis one-way analysis of variance, otherwise at p < 0.05.

## Results

The demographic, clinical and laboratory data of the groups are shown in Table 1. There were no statistically significant differences between the groups with respect to age and gender (both p > 0.05). Dialysis duration was comparable between HD subjects with or without HCV infection (P > 0.05). Serum uric acid, BUN and creatinine levels were significantly higher in HD subjects with or without HCV infection than controls (all p < 0.05). There was no statistically significant difference between HD subjects with or without HCV infection in respect to serum creatinine, urea and uric acid level (all p > 0.05). ALT levels were comparable between HD subjects with and without HCV infection and controls (all p > 0.05). No correlation was observed between ALT and HCV-RNA level in HCV (+) HD subjects (p > 0.05).

**Table 1:** Clinical and demographic characteristics of the study groups.

**Study Groups**	**HCV (+) HD subjects**	**HCV (-) HD subjects**	**Control subjects**
**N**	16	24	24
**Age (year)**	50.3 ± 10	46.8 ± 8.5	40.4 ± 7.3
**Gender (M/F)**	10/6	13/11	14/10
**Uric Acid (mg/dL)**	5.5 ± 1.5*	5.54 ± 1.25*	4.4 ± 0.3
			
**BUN (mg/dL)**	61 ± 23*	65 ± 31*	16 ± 5.5
			
**ALT**	27 ± 13.7	22.4 ± 10.7	23.3 ± 4.8
**Creatinine (mg/dL)**	6.05 ± 2.4*	7.01 ± 1.5*	0.97 ± 0.15
			
**HCV-RNA (log copies/mL**)	4.9 ± 0.7	--	--
**DD (Years)**	4.25 ± 2.5	3.35 ± 2.7	--
			
**TAC ( mmol Trolox eq./L**)	1.27 ± 0.2	1.68 ± 0.4	2.1 ± 0.6**
**TP (micromole H2O2/L)**	12.9 ± 6.1**	10.9 ± 6.9**	5.2 ± 2.1
**OSI (%)**	1.6 ± 0.6**	1.1 ± 0.9**	0.3 ± 0.1

Data were presented as mean ± SD.

*p < 0.05 vs. controls.

**p < 0.05/3 vs. HD subjects with or without HCV infection.

***p < 0.05/3 vs. controls.

M, male; F, female; DD, dialysis duration; BUN, blood urea nitrogen; HD, hemodialysis;

HCV, hepatitis C virus; ALT, alanine aminotransferase; TAC, total antioxidant capacity; TP, total peroxide level; OSI, oxidative stress index.

**Figure 1:**
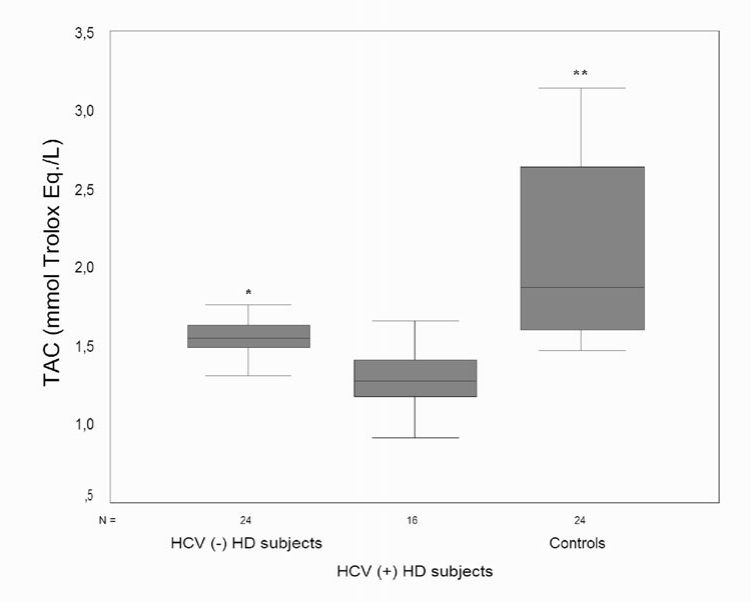
HCV (+) HD subjects had lower TAC than HCV (-) HD subjects. Both HD subjects with or without HCV infection had also lower TAC than controls. TAC, total antioxidant capacity; HCV, hepatitis C; HD, hemodialysis. *p < 0.05/3 vs. HC (+) HD subjects. **p < 0.05/3 vs. HD subjects with or without HCV infection.

**Figure 2:**
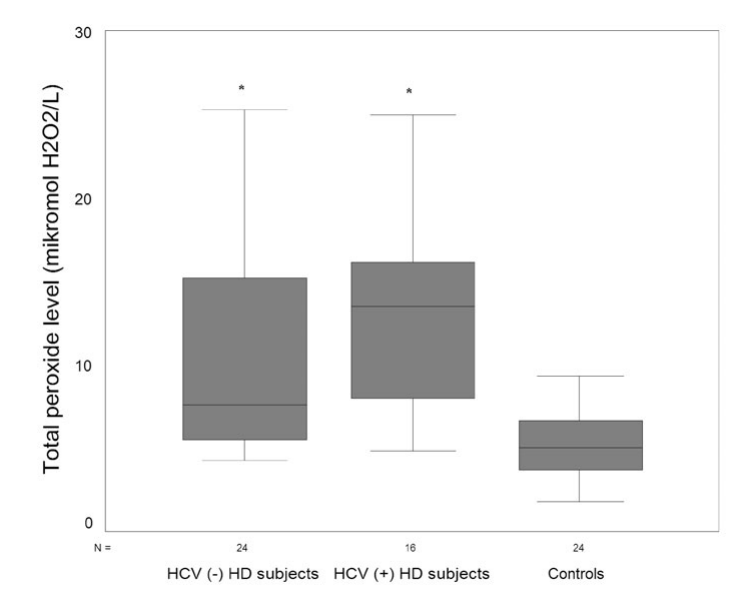
Both HD subjects with or without HCV infection had higher total peroxide level than controls. However, total peroxide level was comparable between HD subjects with or without HCV infection. HCV, hepatitis C; HD, hemodialysis. *p < 0.05/3 vs. controls.

**Figure 3:**
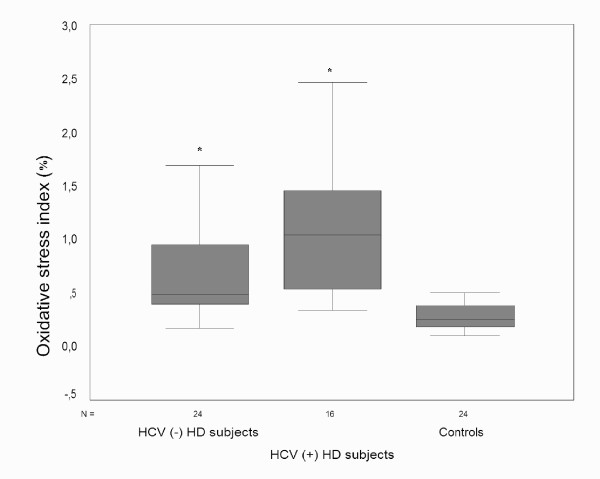
Both HD subjects with or without HCV infection had higher oxidative stress index than controls. However, oxidative stress index was comparable between HD subjects with or without HCV infection. HCV, hepatitis C; HD, hemodialysis. *p < 0.05/3 vs. controls.

TAC was significantly higher in controls than HD subjects with or without HCV infection (all p < 0.05/3), while total peroxide level and OSI were significantly lower (all p < 0.05/3). HCV (-) HD subjects had higher TAC compared to HCV (+) HD subjects (all p < 0.05/3). Total peroxide level and OSI was comparable between HD subjects with or without HCV infection (p > 0.05/3) (Figure 1, 2, 3).

In multiple linear regression analysis, TAC, total peroxide level or OSI were not correlated with age, gender, dialysis duration, HCV positivity, and serum uric acid, BUN and creatinine level (all p > 0.05).

## Discussion

Increased oxidative damage due to reactive oxygen species has been reported in HD subjects [8,9]. Several potential sources such as increase in neutrophil free radical production during HD [24], iron overload [25], reduction of antioxidant enzyme activity due to advanced glycation end products (AGEP)-induced posttranslational modification [26] have been suggested to be responsible from increased radical production in CRF. The increase in free radical formation, manifested by increased hepatic and serum levels of lipid peroxidation products [14,15], and decreased antioxidant levels [27] have also been reported in subjects with HCV infection.

In the previous studies, which were conducted in HCV (+) HD subjects, the oxidative status of subjects was determined using measurement of pentosidine level [17], thioredoxin level [18] or both malondialdehyde (MDA), protein carbonyl content and protein sulfhydryl groups [16]. It is well known that, the effects of various antioxidants in plasma are additive and the cooperation of antioxidants in human serum provides protection of the organism against attacks by free radicals [28]. Therefore, the measurement of TAC may reflect accurately the antioxidant status of the organism [1,19]. Although determination of either oxidants or antioxidant components alone may give information about the oxidative stress, determination of oxidants along with antioxidants is more useful in this context. Therefore, oxidants and anti-oxidant capacity should be measured simultaneously to assess oxidative stress more exactly. In addition, the ratio percentage of the total plasma peroxide level to TAC, regarded as OSI, an indicator of oxidative stress, reflects the redox balance between oxidation and antioxidation [19,20]. Although, in those previous studies [16-18], HCV (+) HD subjects have been reported to be under higher oxidative stress compared to HCV (-) HD subjects, TAC was not determined and OSI was not calculated. Thus, this is the first study determining oxidative status of HD subjects with or without HCV infection using measurement of TAC along with measurement of total peroxide level and calculation of OSI.

In the present study, if only total antioxidant capacity measurement results were taken into account, HCV (+) HD subjects might be falsely assumed to have aggravated oxidative stress compared to HCV (-) HD subjects. However, total peroxide level and OSI, which reflects the redox balance between oxidation and antioxidation, showed no difference between those groups. On the basis of these findings, it can be suggested that HCV infection has no additional influence on oxidative status in HD subjects.

Since each one of ESRD and HCV infection increases oxidative stress, it is logical to expect that HCV (+) HD subjects may be under higher oxidative stress than HCV (-) HD subjects. It has been shown that HCV viral load in HD population with HCV infection is typically low and suggested that the HD procedure lowers HCV RNA levels by various mechanisms: the clearance of HCV RNA by the dialysate, the entrapment of HCV RNA particles onto the membrane surface of dialyser, and the production of cytokines and other substance during the HD session [29].

Although, in HD subjects, HCV-related liver disease usually runs an indolent course [13], the mortality of HCV infected HD patients seems to be enhanced compared with HCV (-) HD patients and it is mainly due to hepatocellular carcinoma and liver cirrhosis [30,31]. It has been suggested that increased oxidative stress is a possible risk factor contributing to development of DNA damage, which may be associated with various cancers and cardiovascular disease [32]. However, in accordance with our observation, there is no data reflecting additional increase in oxidative stress, and consequently in DNA damage among HCV (+) HD subjects. Although the incidence of liver cancer among HCV (+) HD subjects has been reported to be significantly higher than HCV (-) HD subjects [33], it seems equal to the reported incidence of liver cancer among HCV (+) subjects without renal disease [34-36]. In addition, there is no data reporting increased incidence of the other disorders such as other cancers, cardiovascular and neurodegenerative diseases, which might be associated with increased oxidative stress, and consequently in DNA damage among HCV (+) HD subjects compared to HCV (-) HD subjects.

In the present study, in respect to ALT levels, no significant difference was observed between HD subjects with or without HCV infection and controls. It has been reported that ALT levels in anti-HCV positive subjects on maintenance dialysis are often in range considered normal for the general population [37]. In this regard; our finding was consistent with the literature. Although the precise role of viral, host and/or environmental factors in promoting disease progression have yet to be defined in dialysis subjects, it has been reported that inflammatory cytokine responses such as production of tumor necrosis factor (TNF)-alpha, interleukin (IL-) 6, IL-10, interferon (IFN-) gamma by blood mononuclear cells were abolished in hemodialysis patients. The pattern of response reported to be similar among hemodialysis patients with or without HCV infection. In addition, it has been shown that HCV-positive hemodialysis patients had a blunted TNF-alpha response and failed to increase the stimulated IFN-gamma and IL-12 production compared with chronic hepatitis C patients without renal disease [38]. Therefore, both the protective activity of hemodialysis on the course of HCV infection and attenuated inflammatory reactions in the liver due to reduced immune competence of chronic uremic patients may be plausible factors reducing hepatocyte destruction, which reflected by the ALT levels.

Our study has several shortcomings that need to be stressed. Firstly, the oxidative stress assessment was based on single determination of plasma peroxide concentration. A sequential measurement could be necessary to avoid possible plasma fluctuations of peroxide concentartion Secondly, only one oxidative stress marker was used to portrait oxidative status. Other oxidative stress markers which are recognizably elevated in HD population such as markers of protein, lipids and DNA damage were not measured. Finally, inflammatory markers which are well known as strong predictor of mortality were not provided.

In conclusion, in the lightening of our findings, it can be suggested that oxidative stress is increased in both HCV (+) and HCV (-) HD subjects. However, HCV infection seems to not cause any additional increase in oxidative stress in HD subjects and it may be partly due to protective effect of dialysis treatment on HCV infection.

## Abbreviations

CRF, chronic renal failure; HD, hemodialysis; PD, peritoneal dialysis; HCV, hepatitis C virus; ALT, alanine-aminotransferase; TAC, total antioxidant capacity; OSI, oxidative stress index; ESRD, end-stage renal disease; IV, intravenous; MEIA, micro particle enzyme immunoassay; RT-PCR, real time polymerase chain reaction; BUN, blood urea nitrogen; FOX, ferrous oxidation in xylenol orange; BHT, butylated hydroxytoluene.

## Competing interests

The author(s) declare that they have no competing interests.

## Authors' contributions

MH, CB, FFB, AOK, MA: Conception and design; MH, CB, SS, OE: Analysis and interpretation of the data; MH, CB, FFB, MA, AOK: Drafting of the article; MH, CB, FFB, OE, HC: Critical revision of the article for important intellectual content; MH, CB, FFB, MA, AOK, SS, OE: provision of study materials or patients; MH, CB: Statistical expertise; MA, AOK, SS, OE, HC: Collection and assembly of data. All authors read and approved the final manuscript.

## Pre-publication history

The pre-publication history for this paper can be accessed here:


